# CRISPR-Mediated Reactivation of DKK3 Expression Attenuates TGF-β Signaling in Prostate Cancer

**DOI:** 10.3390/cancers10060165

**Published:** 2018-05-28

**Authors:** Hoda Kardooni, Estela Gonzalez-Gualda, Emmanouil Stylianakis, Sina Saffaran, Jonathan Waxman, Robert M. Kypta

**Affiliations:** 1 Department of Surgery and Cancer, Imperial College London, London W12 0NN, UK; h.kardooni@imperial.ac.uk (H.K.); eg460@cam.ac.uk (E.G.-G.); e.stylianakis17@imperial.ac.uk (E.S.); j.waxman@imperial.ac.uk (J.W.); 2 School of Engineering, University of Warwick, Coventry CV4 7AL, UK; S.Saffaran.1@warwick.ac.uk; 3 Centre for Cooperative Research in Biosciences, CIC bioGUNE, 48160 Derio, Spain

**Keywords:** prostate cancer, gene promoter methylation, TGF-β signaling, Dickkopf-3, PTGS2

## Abstract

The *DKK3* gene encodes a secreted protein, Dkk-3, that inhibits prostate tumor growth and metastasis. *DKK3* is downregulated by promoter methylation in many types of cancer, including prostate cancer. Gene silencing studies have shown that Dkk-3 maintains normal prostate epithelial cell homeostasis by limiting TGF-β/Smad signaling. While ectopic expression of Dkk-3 leads to prostate cancer cell apoptosis, it is unclear if Dkk-3 has a physiological role in cancer cells. Here, we show that treatment of PC3 prostate cancer cells with the DNA methyltransferase (DNMT) inhibitor decitabine demethylates the *DKK3* promoter, induces DKK3 expression, and inhibits TGF-β/Smad-dependent transcriptional activity. Direct induction of DKK3 expression using CRISPR-dCas9-VPR also inhibited TGF-β/Smad-dependent transcription and attenuated PC3 cell migration and proliferation. These effects were not observed in C4-2B cells, which do not respond to TGF-β. TGF-β signals can regulate gene expression directly via SMAD proteins and indirectly by increasing DNMT expression, leading to promoter methylation. Analysis of genes downregulated by promoter methylation and predicted to be regulated by TGF-β found that DKK3 induction increased expression of PTGS2, which encodes cyclooxygenase-2. Together, these observations provide support for using CRISPR-mediated induction of DKK3 as a potential therapeutic approach for prostate cancer and highlight complexities in Dkk-3 regulation of TGF-β signaling.

## 1. Introduction

Prostate cancer (PCa) is the most commonly diagnosed cancer and the second cause of cancer-related deaths among men in the U.S. [1]. Survival rates in the last five years have improved as a result of improved earlier diagnosis and the implementation of prostate specific antigen (PSA) test screening. First line treatment includes androgen receptor (AR)-directed therapies, since most tumors express AR and depend on its signaling. However, many patient tumors become resistant after hormone-therapy and relapse within 24 months, developing castration-resistant PCa (CRPC) [2], highlighting the need for novel therapies and treatments targeting alternative signaling pathways involved in PCa progression. Recent studies have demonstrated that epigenetic alterations, particularly changes in gene promoter methylation, are frequent during PCa tumorigenesis [3]. Cancer genomics studies have identified recurrently mutated genes and mutation hotspots in a number of cancer types. However, such studies in prostate adenocarcinomas have identified no genes recurrently mutated in more than a seventh of cases [4]. The biological, and hence clinical behavior, of prostate adenocarcinomas may therefore be determined by variations in the levels of expression of a range of genes, regulated by epigenetic mechanisms. Epigenetic aberrations play an important role in the pathogenesis of the majority of cancers, and one of the most widespread and powerful mechanisms is gene silencing through DNA methylation [5]. DNA methylation is a chemical process defined as the covalent addition of a methyl group to the 5′ carbon of a cytosine nucleotide, leading to the formation of 5-methylcytosine within the so-called CpG dinucleotides [6]. CpG dinucleotides are usually located in clusters named CpG islands, commonly found in the promoter regions of genes, and their hypermethylation results in the repression of gene transcription by further direct and indirect mechanisms [5,7,8]. The detection of DNA methylation changes in cancer genomes can highlight genes that may provide evidence of deregulated signaling networks and pathways, as well as potential candidates for biomarker development. The process of DNA methylation is tightly regulated by DNA methyltransferases (DNMTs) and DNA demethylases, respectively [9]. The DNMT family includes DNMT1, DNMT3 (DNMT3A, DNMT3B), and DNMT3L [8]. DNMT3A and DNMT3B are de novo methyltransferases that function independently of replication and initiate methylation at CpG sites on unmethylated and/or hemi-methylated DNA [10]. On the other hand, DNMT1 is a maintenance methyltransferase that acts during replication and has a preference for hemi-methylated DNA [11]. Demethylation is carried out by the ten-eleven translocation (TET) family of DNA hydroxylases, which regulate demethylation by oxidizing 5-methylcytosine to 5-hydroxymethylcytosine and further derivatives and are deregulated in many cancer, including prostate cancer [12].

To date, over 860 genes have been described as hypermethylated in PCa [3]. Of note, the Dickkopf-related protein 3 (DKK3) gene, also known as ‘reduced expression in immortalized cells’ (REIC), is remarkably silenced by promoter methylation in many types of cancer, including PCa [13,14,15], particularly in high Gleason grade tumors [16]. The DKK3 gene product, Dickkopf-3 (Dkk-3), plays a role in prostate gland architecture [17] and displays potent tumor suppressor activity [18]. Ectopic expression of DKK3 in PCa cells leads to apoptosis [13,19] and inhibits proliferation, migration [15,20], and metastasis [19]. Moreover, injection of a DKK3-expressing adenovirus into the tumors of a patient with incurable PCa reduced metastatic tumor growth [21], and an early stage clinical trial found that intra-prostatic injection of DKK3 adenovirus significantly improved survival of patients with high-risk localized PCa [22]. Thus, it may be possible to develop new therapies that re-activate Dkk-3 expression or that activate the Dkk-3 signal in cancer cells.

Dkk-3 is a member of a family of secreted glycoproteins first identified as inhibitors of Wnt/β-catenin signaling, through their ability to bind to LRP5/6 Wnt co-receptors [23]. Unlike the other family members, however, Dkk-3 does not associate with LRP5/6 and its effect on Wnt/β-catenin activity remains controversial [21]. We and others have found that Dkk-3 regulates the response to transforming growth factor-β (TGF-β) in prostate epithelial, stromal, and PCa cells [20,24,25]. TGF-β signaling can have pro- and anti-tumorigenic effects, depending on cellular context and stage of progression [26]. Binding of TGF-β to its receptors leads to phosphorylation of Smad2/3, resulting in transcriptional regulation of target genes. In early-stage cancers, this leads to cell cycle arrest and/or apoptosis, but is pro-tumorigenic in advanced cancers, promoting tumor cell motility, invasion, and metastasis [27]. The molecular drivers for the switch from anti- to pro-tumorigenic effects are attractive targets for cancer drug development and are under intense research [28,29,30,31]. In addition, TGF-β increases DNMT expression in PCa [32], providing an indirect route to regulation of gene expression. Indeed, many genes found to be hypermethylated in PCa are linked to TGF-β signaling [33].

A number of DNA demethylating drugs have been approved by the FDA (Food and Drug Administration), for example, azacitidine and decitabine [34]. Despite positive responses in acute myeloid leukemia patients, acquired resistance is common, and these drugs are less effective in solid tumors. Improved versions are being developed and are in clinical trials for PCa (reviewed in [35] [36]), but this class of inhibitors will always have a global impact on the epigenome and therefore lack specificity for tumor suppressor genes. Thus, alternative methods targeting specific epigenetic alterations in cancer may be a more effective approach. In this context, the CRISPR-Cas9 system is attractive, as it can be used to target genes precisely. CRISPR-Cas9 genome editing uses the endonuclease Cas9 to induce targeted indels and precise sequence changes. A guide RNA (gRNA) molecule binds to Cas9 and directs it to a complementary target sequence, where the protein performs a DNA double-stranded break. Several groups have developed modified versions of Cas9 for applications that go beyond genome editing. Bikard et al*.* engineered dCas9 by mutating the Cas9 catalytic site [37], and Chavez et al. fused dCas9 to a tripartite transcriptional activation domain (dCas9-VPR) to induce expression of gRNA target genes [38]. Here, we use dCas9-VPR and gRNAs targeting the DKK3 gene promoter to examine the consequences of re-activating endogenous DKK3 expression on PCa cell physiology and on the expression of genes regulated by promoter methylation. Our results demonstrate that CRISPR-dCas9-VPR induction of Dkk-3 is sufficient to inhibit the response to TGF-β and alter the expression of PTGS2, a TGF-β regulated gene that is methylated in PCa.

## 2. Results

### 2.1. Decitabine Treatment of PC3 Cells Increases DKK3 Expression and Inhibits TGF-β-Dependent Gene Reporter Activity

**Figure 1:**
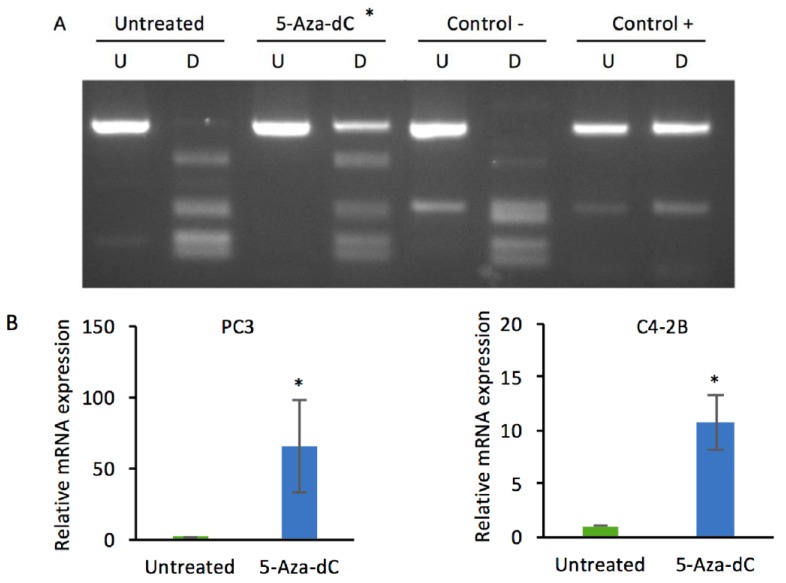

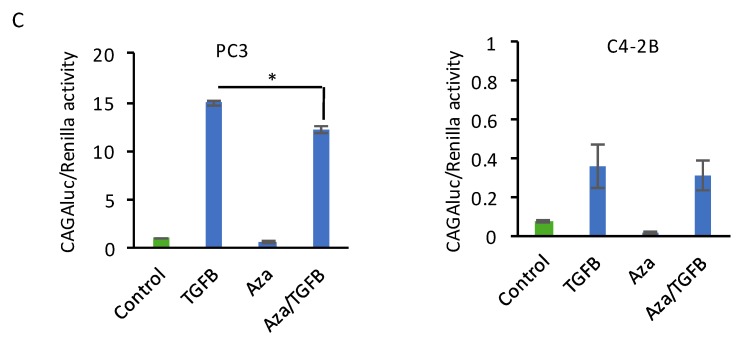
Decitabine treatment of PC3 cells increases DKK3 expression and inhibits TGF-β-dependent gene reporter activity. (**A**) Combined bisulfite restriction analysis (CoBRA) of *DKK3* gene promoter methylation in PC3 cells after treatment with decitabine (2 µM, 3 days); gel shows undigested (U) and BstUI-digested (D) PCR products, * promoter de-methylation. (**B**) Q-RT-PCR analysis of *DKK3* mRNA levels in untreated and 5-aza-2′deoxycytidine (5-aza-dC)-treated PC3 and C4-2B cells; * *p* < 0.05, 2-tailed Student’s *t*-test versus untreated. (**C**) TGF-β-dependent gene reporter activity (luciferase/renilla), relative to control, in PC3 and C4-2B cells transfected with CAGA12-luciferase and renilla and either untreated (control) or treated with decitabine (Aza) and/or TGF-β for 24 h. Data are shown as means ± SEM (*n* = 3). * *p* < 0.05, ANOVA and two-tailed Student’s *t*-test.

In order to confirm that *DKK3* mRNA expression is repressed by promoter methylation, PC3 cells were analyzed using Combined Bisulfite Restriction Analysis (CoBRA) to determine the extent of *DKK3* gene methylation at the promoter CpG island. This confirmed a high level of methylation that was reduced by treatment of cells with decitabine (Figure 1A), consistent with previous studies [39]. Moreover, analysis of DKK3 gene expression by q-RT-PCR indicated that decitabine treatment increased *DKK3* mRNA levels, both in PC3 cells, as previously reported [39], and in C4-2B cells (Figure 1B). Given the links between Dkk-3 and TGF-β-signaling [20,24,40], gene reporter assays were carried out to determine the effects of decitabine on TGF-β-dependent transcription using pGL3-CAGA12, which encodes the luciferase gene fused to 12 repeats of a Smad-binding site, and renilla, to control for transfection efficiency. Decitabine reduced TGF-β-dependent gene reporter activity in PC3 cells but not in C4-2B cells (Figure 1C), consistent with Dkk-3 inhibition of TGF-β signaling in PC3 cells and not in C4-2B cells, which do not express TGFBR2 [41].

### 2.2. CRISPR-Mediated Activation of the DKK3 Promoter Increases DKK3 mRNA and Dkk-3 Protein Levels

**Figure 2:**
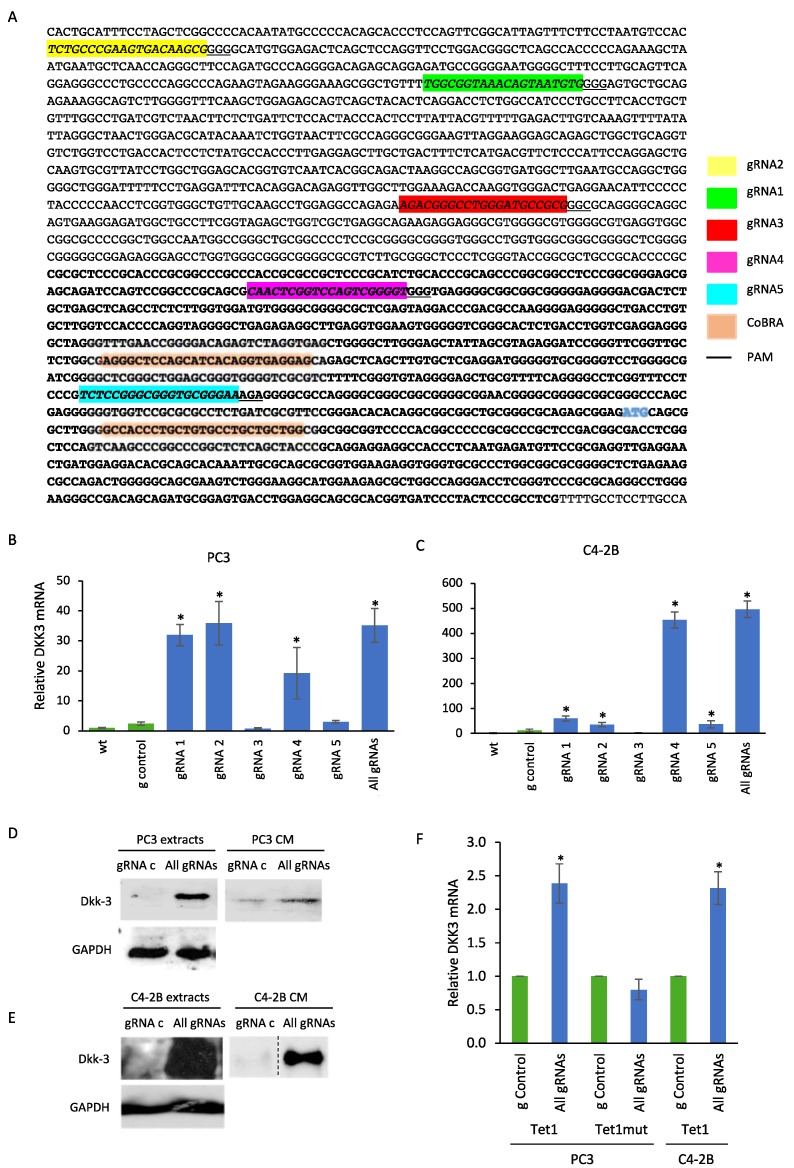
CRISPR-mediated induction of DKK3. (**A**) DKK3 gene promoter with the positions of gRNAs, PAM sequences (underlined) and translation start (blue). (**B**,**C**) Q-RT-PCR analysis of DKK3 mRNA expression in PC3 (**B**) and C4-2B (**C**) cells 48 h after transfection with dCas9-VPR and the indicated gRNAs; graphs show mean ± SD of triplicate wells, *n* = 5. * *p* < 0.05 versus control gRNA, one-way ANOVA and two-tailed Student’s *t*-test. (**D**,**E**) Western blots of cell lysates (extracts) and conditioned media (CM) from PC3 (**D**) and C4-2B (**E**) cells 5 d after transfection with dCas9-VPR and control gRNA (gRNA-C) or DKK3 gRNAs 1, 2, 3, 4 and 5 together (All-gRNAs) and treated for the first two days with 1 μg/mL puromycin, were probed for Dkk-3 and GAPDH. (**F**) DKK3 mRNA expression in PC3 and C4-2B cells after transfection with dCas9-Tet1CD (Tet1) or the inactive mutant (Tet1mut); graphs show mean ± SD of triplicate wells, *n* = 4; * *p* < 0.05 versus control gRNA**,** two-tailed Student’s *t*-test.

Decitabine is likely to have global effects on gene expression, so its effect on TGF-β-dependent gene reporter activity could be unrelated to *DKK3* mRNA expression. Therefore, in order to activate endogenous DKK3 expression specifically, we used CRISPR to target the transcriptional activator dCas9-VPR to the DKK3 gene promoter. Five guide RNAs (gRNAs) targeting different sites of the DKK3 promoter were designed (Figure 2A). Plasmids expressing these gRNAs, individually or in combination were co-transfected with dCas9-VPR plasmid and DKK3 mRNA expression was measured by q-RT-PCR after 48 h. The results showed a remarkable increase of *DKK3* mRNA levels, the extent of which depended on the gRNA used and the cell line transfected. In PC3 cells, gRNA-1, -2 and -4 significantly increased DKK3 gene expression, as did the combination of all five gRNAs (gAll) (Figure 2B). The same gRNAs increased DKK3 expression in C4-2B cells, with gRNA-4 being the most effective, achieving a >400-fold increase (Figure 2C). Next, western blotting analysis was used to determine if the increases in *DKK3* mRNA expression were sufficient to lead to detectable levels of Dkk-3 protein. Analysis of cell extracts and cell-conditioned media (CM) five days after transfection revealed that transfection of dCas9-VPR with the combination of all five gRNAs led to detectable levels of Dkk-3 protein in cell extracts and in cell CM, both in PC3 (Figure 2D) and C4-2B (Figure 2E) cells. ELISA analysis of cell CM at 72 h found that the amounts of Dkk-3 in CM from PC3 cells transfected with control gRNA and DKK3 gRNAs were 0.027 ± 0.033 and 0.105 ± 0.084 ng/mL (mean and SD, *n* = 3), respectively. In C4-2B cell CM, this was 0.035 and 1.3 ng/ml, respectively. These results indicate that CRISPR/dCas9-VPR induction of *DKK3* mRNA expression leads to low but measurable levels of secretion of Dkk-3 protein. In order to determine if DKK3 expression could be induced by targeted demethylation of the *DKK3* promoter, we used an expression vector encoding dCas9 fused to the catalytic domain of the demethylase Tet1 (dCas9-Tet1CD) [42]. Expression of DKK3 gRNAs with dCas9-Tet1CD significantly increased *DKK3* mRNA expression in PC3 cells and C4-2B cells, and this was not observed using an inactive mutant of Tet1 (Figure 2F). However, we were unable to detect Dkk-3 protein at the time point used in either cell line.

### 2.3. CRISPR-Mediated Induction of Dkk-3 Inhibits TGF-β Signaling in PC3 Cells

**Figure 3:**
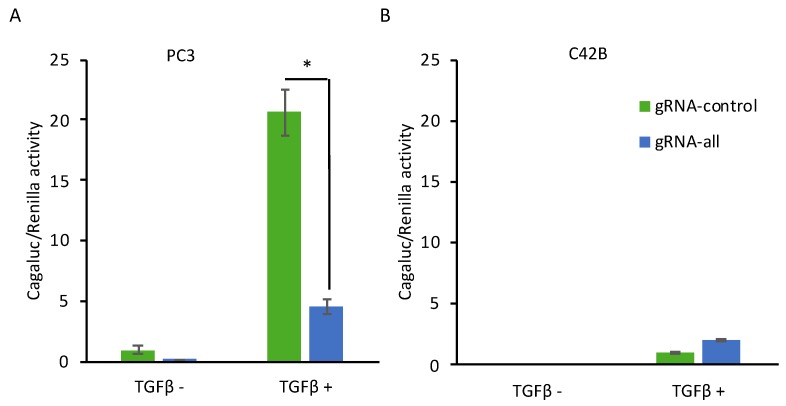
CRISPR-mediated induction of Dkk-3 inhibits TGF-β signaling in PC3 cells. (**A**,**B**) Gene reporter assays using PC3 (**A**) and C4-2B (**B**) cells transfected with CAGA12-luciferase, renilla, dCas9-VPR and either gRNA-control (gRNA-C) or DKK3 gRNAs (gRNA-all). TGF-β1 (2 ng/mL) was added 24 h after transfection and reporter activity was measured after 24 h. Graphs show mean CAGA luciferase/renilla activity, relative to untreated gRNA-control cells. * *p* < 0.05, two-tailed Student’s *t*-test, *n* = 3.

In order to determine if CRISPR induction of Dkk-3 inhibits TGF-β-dependent signaling, gene reporter assays were carried out using cells transfected with luciferase reporters and dCas9-VPR and either control or DKK3 gRNAs. TGF-β treatment increased CAGA luciferase activity in PC3 cells transfected with control gRNA, as expected (Figure 3A). This activity was reduced by transfection of DKK3 gRNAs, indicating that CRISPR-mediated induction of Dkk-3 inhibits TGF-β-dependent signaling. In contrast, induction of Dkk-3 in C4-2B cells had no effect on CAGA luciferase activity (Figure 3B), consistent with the negligible effects of TGF-β in these cells, which do not express TGF-β receptors [41].

### 2.4. CRISPR-Mediated Induction of Dkk-3 Reduces PC3 Cell Number

**Figure 4:**
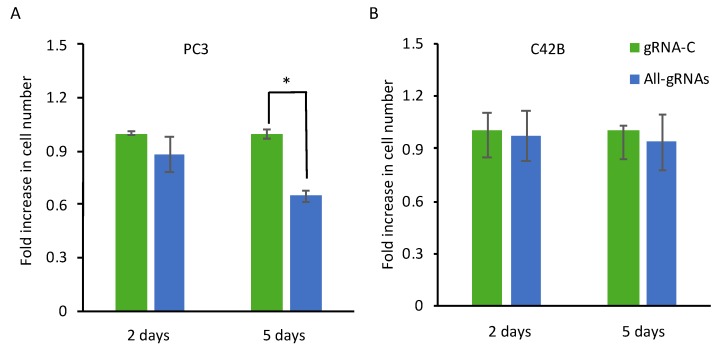
CRISPR-mediated induction of Dkk-3 reduces PC3 but not C4-2B cell number. (**A**,**B**) Crystal violet assays for PC3 (**A**) and C4-2B (**B**) cells transfected with dCas9-VPR and control (gRNA-C) or DKK3 (All-gRNAs) gRNAs after two or five days. Values shown represent the average fold change, relative to gRNA-C cells at each time point, ± SD of triplicate wells (*n* = 3). * *p* < 0.05, two-tailed Student’s *t*-test.

Ectopic expression of Dkk-3 rapidly induces apoptosis in prostate cancer cells [13]. To determine if CRISPR-mediated induction of DKK3 affects prostate cancer cell number, cells were transfected with dCas9-VPR and control or DKK3 gRNAs and cultured for two days and five days, after which cells were fixed and stained using crystal violet to provide a measure of cell number. Compared to control gRNA, transfection of dCas9-VPR with DKK3 gRNAs did not significantly affect cell number at two days (Figure 4A). After five days, however, there was a notable and significant reduction in cell number. On the other hand, there was no effect on C4-2B cell number at either time point (Figure 4B). These results indicate that CRISPR activation of DKK3 expression has a limited and delayed effect on cell number, in contrast to what has been observed using ectopically expressed DKK3.

### 2.5. CRISPR-Mediated Induction of Dkk-3 Reduces PC3 Cell Migration

**Figure 5:**
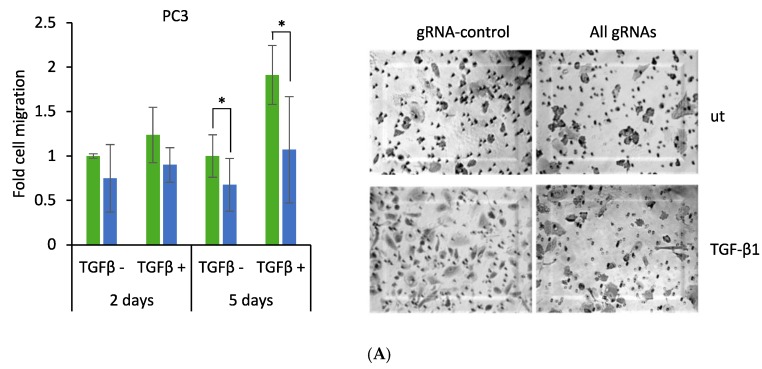

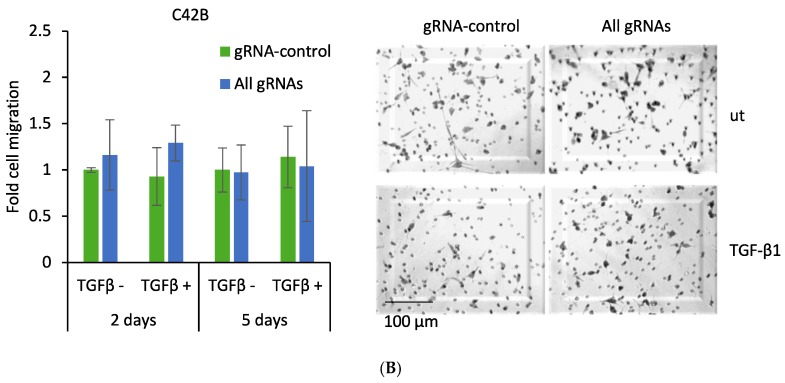
CRISPR-mediated induction of Dkk-3 reduces PC3 cell migration. PC3 and C4-2B cells transfected for one or four days with dCas9-VPR and either control (gRNA-control) or DKK3 (All gRNAs) gRNAs were plated in transwell inserts in serum-free medium ± 2 ng/mL TGF-β1 and allowed to migrate for 24 h. (**A**,**B**) Graphs show average fold change in cell migration (± TGF-β) for PC3 (**A**) and C4-2B (**B**) cells, 2 d or 5 d after transfection, relative to migration of untreated gRNA-control cells, ± SD of duplicate wells (*n* = 3). * *p* < 0.05, two-tailed Student’s *t*-test. Representative images (5d) show migrated cells in the inserts stained with crystal violet, 200× magnification.

Given the effect of CRISPR induction of DKK3 on TGF-β-dependent transcriptional activity, we wished to determine if it also had an impact on TGF-β-dependent prostate cancer cell migration. To this end, cells were transfected, cultured for one or four days and then plated in inserts in serum-free media with or without TGF-β, and with medium containing 10% FCS in the lower chamber. After 24 h, migrated cells were stained and counted. CRISPR induction of DKK3 did not significantly affect the migration of PC3 (Figure 5A) or C4-2B (Figure 5B) cells when they were cultured for one day prior to plating for migration assays. However, when transfected cells were cultured for four days prior to plating, expression of dCas9-VPR and DKK3 gRNAs significantly reduced TGF-β-dependent migration (Figure 5A). A reduction was also observed both in the absence of exogenous TGF-β, possibly reflecting inhibition of autocrine TGF-β signaling. In contrast, there was no significant effect on C4-2B cell migration (Figure 5B). Equal numbers of viable cells were plated in each case, so the effect on cell migration was not a result of a reduction in cell number. These results indicate that activation of endogenous DKK3 expression inhibits PC3 but not C4-2B cell migration, in keeping with its effects on TGF-β signaling in these cell lines.

### 2.6. Effect of Activation of Endogenous DKK3 on Gene Expression

**Figure 6:**
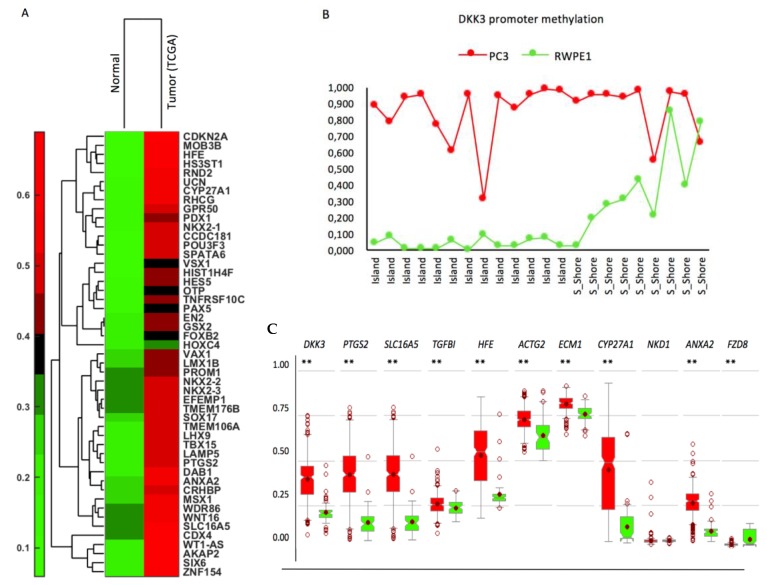
Hypermethylation of the DKK3 promoter region (**A**) Dendrogram of two-dimensional hierarchical clustering analysis of 49 genes that are more highly methylated in tumor samples than in normal prostate, selected by comparison of average promoter methylation (beta-values) in multiple studies of prostate. (**B**) Average methylation (beta-values) of DKK3 promoter region in PC3 and RWPE1 cells from GEO (GSM1323600 and GSM1323601, respectively). (**C**) Methylation status (percentage) in the CpG sites of the promoter regions for the indicated genes in normal prostate and prostate cancer, extracted from the TGCA dataset using the MethHC database.

TGF-β regulates the expression of DNMT1, whose expression predicts disease recurrence [32], and many genes hypermethylated in PCa are linked to TGF-β signaling [33]. We therefore wished to determine if DKK3 has an impact on genes that are methylated in PCa and potentially regulated by TGF-β. To identify such genes, we re-analyzed data for differential DNA methylation of genes in PCa and normal (benign) prostate [3], focusing on the 168 genes that were identified as hypermethylated in at least 3 of the 17 studies examined (Figure S1). Defining probes with β-values > 0.5 and < 0.35 as methylated and un-methylated, respectively, provided a list of 49 genes that were differentially methylated in benign prostate and PCa (Figure 6A**)**. The differential mRNA expression of these genes in PCa, compared to in normal prostate was then analyzed using PCa datasets from Oncomine (https://www.oncomine.org) and cBioPortal for Cancer Genomics (http://www.cbioportal.org) and the genes were ranked according to their downregulated expression in PCa (Figure S2). Finally, ToppGene (https://toppgene.cchmc.org/) was used to identify genes with significant links to TGF-β signaling. Among these genes, we selected *PTSG2*, *ANXA2*, *HFE*, *SLC16A5*, and *CYP27A1* for further analysis. In addition, we analyzed *DKK3* and other genes potentially regulated by DKK3 [43], by TGF-β (*FZD8* [44]) or by Wnt signaling (*NKD1*).

Analysis of the methylation status of the promoters of these genes in PC3 cells using Gene Expression Omnibus (GEO) (ncbi.nlm.nih.gov/geo/) indicated that *DKK3*, *SLC16A5*, *PTGS2*, *HFE*, and *TGFBI* had β-values > 0.5, while *CYP27A1*, *ANXA2*, and *FZD8* had β-values < 0.35. For the *DKK3* promoter (Figure S3) the average β-values were 0.86 and 0.18 in PC3 and RWPE1 cells, respectively (Figure 6B). Analysis of these genes in patient samples from the TGCA dataset using the MethHC database (methhc.mbc.nctu.edu.tw) confirmed significant hypermethylation of the *DKK3*, *PTGS2*, *SLC16A5*, *HFE*, and *CYP27A1* promoters in prostate tumors, compared to normal prostate. *ACTG2* and *ECM1* were also more highly methylated in tumors than in normal prostate, but from a high basal level, while *TGFBI* and *ANXA2* were more highly methylated in tumors, but from a low basal level in normal prostate. *NKD1* and *FZD8* were only very weakly methylated both in patient tumors and in normal prostate (Figure 6C).

**Figure 7:**
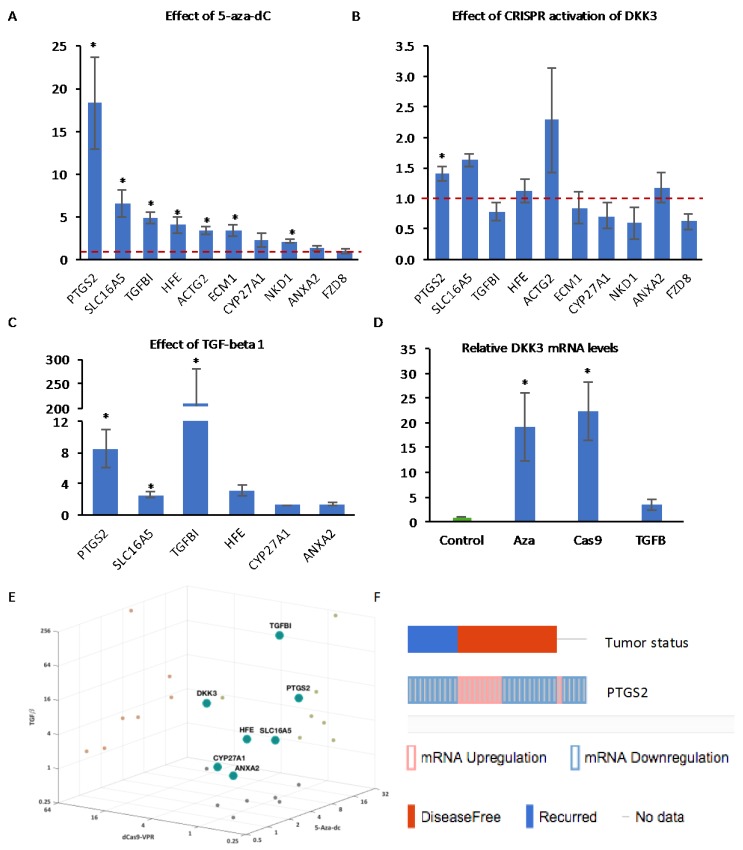
Q-RT-PCR analysis of the expression of the indicated genes in PC3 cells (**A**) treated with 5_Aza-dC for 72 h, (**B**) transfected with dCas9-VPR and control gRNA or DKK3 gRNAs for 48 h and (**C**) treated with TGF-β1 for 24 h. (**D**). Q-RT-PCR analysis of the expression of DKK3 in PC3 cells under the indicated conditions. Graphs show mean expression relative to control (dashed red lines indicate control level set to 1) ± SD, *n* = 3 to 5, * *p* < 0.05 two-tailed Student’s *t*-test. (**E**) 3D plot illustrating the effects of decitabine, CRISPR induction of DKK3 and TGF-β1 on the indicated genes. (**F**) Comparison of tumors with changes in PTGS2 expression (up, red; down, blue) and disease-free status of patients, from cBioPortal using the MSKCC 2010 dataset [45].

In order to determine the extent to which the expression of these genes is regulated by gene promoter methylation in PCa, q-RT-PCR analysis was carried out to measure mRNA expression in PC3 cells treated with decitabine. Most of the genes were significantly upregulated after 24 h, with *PTGS2* being the most highly-induced (Figure 7A), while there was no effect on *ANXA2* and *FZD8*, consistent with their low levels of methylation. We then examined the effects of CRISPR-mediated induction of *DKK3* on the expression of the same genes. Induction of *DKK3* resulted in a significant increase in the expression of *PTGS2* and trends for increased expression of *SLC16A5* and for reduced expression of *TGFBI* and *FZD8* (Figure 7B). Finally, we examined the effects of TGF-β on the expression of some of the genes. As expected, *TGFBI* was highly-induced by TGF-β treatment of PC3 cells (Figure 7C). In addition, TGF-β treatment resulted in significant increases in the expression of *PTGS2* and *SLC16A5* and a trend for increased expression of *HFE*. CRISPR induction of *DKK3* (22-fold) was similar to its induction by decitabine (19-fold), while there was a trend for increased DKK3 expression in TGF-β-treated cells (Figure 7D). Comparison of the effects of decitabine, CRISPR-mediated DKK3 activation, and TGF-β suggested that, among the genes examined, the regulation of *PTGS2* expression matched most closely to that of *DKK3* (Figure 7E). To determine the clinical relevance of *PTGS2* expression, cBioPortal was used to look for correlations with disease recurrence, which found *PTGS2* levels altered >1.5-fold in 24% of the 150 sequenced patient tumors from the Taylor et al. MSKCC dataset [45] (Figure 7F). Importantly, among the recurrent tumors, only reduction in expression was observed (10 cases), whereas among the disease-free tumors, PTGS2 was reduced in 11 and increased in 9 cases.

## 3. Discussion

Epigenetic silencing of the tumor suppressor *DKK3* by promoter methylation is a common event in many solid tumors and is more prevalent in advanced stages of the disease. Advanced PCa continues to have a poor survival rate due to acquired resistance to the treatments currently available, emphasizing the need for finding new targets for more effective therapies. When designing strategies to prevent cancer progression, gene hypermethylation presents the advantage of being readily reversible, in contrast to permanent alterations in DNA, such as point mutations or gene deletions. Considering the previous reports describing the tumor suppressive roles of DKK3 [13,15,19,20], its inhibitory effect on malignant TGF-β signaling [20,24], and its potential in recent clinical trials [21,22], we have focused on reactivating endogenous *DKK3* expression using a CRISPR-based approach. This has advantages over ectopic expression of DKK3, which can be toxic [46], and over using recombinant Dkk-3, which has little apparent activity in many contexts [25]. Moreover, by directly targeting the *DKK3* genomic locus, the CRISPR-based approach should result in expression of all variants. This is particularly important when considering the effects of Dkk-3 isoforms, for example, the intracellular form of Dkk-3, Dkk-3b [47]. CRISPR activation of DKK3 also provides specificity, unlike demethylating drugs [34]. In this study, we used dCas9-VPR to induce DKK3 expression to a level that was sufficient to affect PC3 cell number and migration. The VPR transcriptional activation domain proved essential in this context, since induction of DKK3 mRNA by dCas9-Tet1CD did not lead to increased Dkk-3 protein levels. However, we anticipate that targeted demethylation can be improved by using lentiviral delivery and allowing more time for induction to take place. A general limitation of the CRISPR approach is that it requires a means of delivery, for example using viral vectors (reviewed in [48]). However, a modified CRISPR/Cas9 system was recently used with success for gene activation *in vivo* [49], indicating the emerging importance of this technology in translational applications.

A major goal of this study was to investigate the effect of DKK3 re-expression on TGF-β/Smad signaling, since overexpression of this pathway is frequently observed in advanced metastatic PCa patients and is associated with poor survival. Our results indicate a significant inhibition of the response to TGF-β1 when DKK3 is re-expressed in PC3 cells. This outcome is in agreement with previous studies reporting links between DKK3 and TGF-β signaling [20,24].

One of the tumor suppressive mechanisms described for DKK3 is its inhibition of cell proliferation [18]. Our results using PC3 cells are consistent with these previous studies and indicate that CRISPR activation of DKK3 has a negative effect on cell number. Importantly, although there was a small reduction in cell proliferation two days after transfection, it was only at the later time point when this reduction was significant. This highlights an important aspect of the approach used, in which time is required for induction of endogenous Dkk-3 to reach a level where it has a measurable physiological effect on cells. In contrast to PC3 cells, DKK3 induction had no effect on C4-2B cell number at the time points studied. This might imply a link between the effects of Dkk-3 on prostate cancer cell proliferation and on TGF-β signaling. However, if this is the case, the mechanism remains unclear, as TGF-β does not affect PC3 cell proliferation [50]. In LNCaP cells, the parental line for C4-2B, adenoviral expression of DKK3 reduces CD147 levels, concomitant with a reduction in cell growth [51]. However, we did not find significant differences in the expression of the genes encoding CD147 or its accessory proteins MCT1/4 after CRISPR-mediated induction of DKK3 in PC3 cells [52]. Given the limited effects of Dkk-3 induction on prostate cancer cell proliferation, it would be interesting to explore its effects when used in combination with other therapies. In this context, downregulation of DKK3 gene expression by DNA promoter methylation in non-small cell lung cancer has been reported to increase resistance to docetaxel [53]. Since docetaxel is the standard chemotherapy drug used to treat castrate-resistant prostate cancer, it would be interesting to determine the effects of combining CRISPR-mediated activation of DKK3 with docetaxel and of restoring DKK3 expression in docetaxel-resistant prostate cancer cells.

In addition to the effect on cell proliferation, we demonstrated that endogenous re-expression of DKK3 results in the attenuation of cell migration. In these assays, we also observed that the effect of DKK3 was only substantial at the later time point. Interestingly, we also detected a significant inhibition in migration in the absence of exogenous TGF-β, which may reflect inhibition of migration driven by autocrine TGF-β signals [50]. TGF-β is a well-established promoter of the epithelial-mesenchymal transition (EMT) in prostate epithelial and prostate cancer cells [54]. This response to TGF-β in prostate epithelial cells is, in part, mediated by increased methylation of the E-cadherin gene (CDH1) promoter [55]. Also, as prostate cancer progresses, it can take on a neuroendocrine (NE)-like phenotype. It is possible, therefore, that induction of DKK3 reverses the EMT or NE-like phenotype. However, preliminary analysis of RNA-Seq data from PC3 cells transiently expressing dCas9-VPR and either control gRNA and DKK3 gRNA did not find significant differences in the expression levels of mesenchymal genes (VIM, SNAI1, SNAI2, SNAI3, ZEB1, ZEB2, TWIST1, TWIST2, and CDH2), epithelial genes (CLD1 and CDH1), or neuroendocrine (NE) genes (ENO2 and CHGA), apart from ZEB2, which increased slightly (1.27-fold, *p* = 0.047) [52]. This suggests that Dkk-3 has a different mechanism of action. Alternatively, the lack of effect on EMT/NE genes may be because PC3 cells express very high levels of EMT and NE genes that are difficult to reverse. Future studies will need to address this question in prostate cancer cell lines with stronger epithelial features and that respond to TGF-β.

Despite strong evidence implicating TGF-β signaling in driving the methylation of several genes in PCa and the inhibition of TGF-β signaling by DKK3, we only observed significant differences in the expression of *PTGS2*. It is important to note that these analyses were performed 48 h after transfection, and therefore, if DKK3 inhibits TGF-β signaling indirectly, more significant changes in gene expression may only be detected at later time points. It remains to be determined if PTGS2 upregulation plays a role in the tumor-suppressive effects of DKK3. This may seem unlikely, as *PTGS2* encodes cyclooxygenase-2 (COX-2), which is upregulated in PCa [56]. However, reduced expression of *PTGS2* mRNA by promoter hypermethylation has been reported to be an indicator of poor prognosis in PCa [57] and the COX-2 inhibitor celecoxib does not improve PCa patient survival [58]. Moreover, our analysis also found a correlation between low *PTGS2* expression and tumor recurrence (Figure 7F).

TGFBI, in contrast, is strongly induced by TGF-β and can promote prostate tumor growth and metastasis [59]. DKK3 silencing increases TGFBI levels in prostate epithelial cells [43], so we anticipated that DKK3 re-expression might inhibit *TGFBI* expression in PC3 cells, and a trend for inhibition was observed.

We were intrigued to note that CRISPR activation of *DKK3*, which inhibited TGF-β-dependent gene reporter activity (Figure 3A), had opposite effects on *PTGS2* and *FZD8* expression, despite both *PTGS2* and *FZD8* being induced by TGF-β. Since *PTGS2* but not *FZD8* was upregulated by decitabine (Figure 7A), we considered the possibility that differences in promoter methylation might account for their differential responses to DKK3. The ability of decitabine to function as an indirect inhibitor of TGF-β signaling has been reported in other contexts. For example, decitabine reverses TGF-β-induced EMT in gastric cancer cells [60] and in PC9 non-small cell lung cancer cells [61] by reducing aberrant TGF-β-induced hypermethylation of the miR-200 gene promoter. At low doses, decitabine induces the proteasomal degradation of DNMT1, reducing proliferation and inducing differentiation [62]. Thus, decitabine might be described as an indirect inhibitor of TGF-β signaling.

TGF-β has been reported to increase DNMT gene expression in prostate cancer [32], which could lead to induction of genes via promoter demethylation. Analysis of *DNMT* family gene expression in PC3 cells confirmed that TGF-β treatment increased expression of DNMT1, DNMT3A, and DNMT3B (Figure S4A). Interestingly, CRISPR induction of DKK3 resulted in a small but significant reduction in the expression of DNMT1 and trends for increased expression of DNMT3A and DNMT3B (Figure S4B). DNMT1 was the most abundant of the DNMTs (average Ct values for DNMT1, DNMT3A, and DNMT3B were approximately 24, 28, and 28, respectively). Thus, by reducing *DNMT1* expression, DKK3 may facilitate promoter demethylation, resulting in increased expression of *PTGS2*, whose expression was found to be most sensitive to decitabine (Figure 7A). In contrast, DKK3 inhibited *FZD8* expression, an unmethylated TGF-β-regulated gene. A limitation of this study is the selection of a small number of genes for analysis. Future studies using whole-genome approaches to measure the effects of DKK3 on gene promoter methylation and transcription should help test this hypothesis and take us closer to determining the mechanism of action of DKK3.

## 4. Materials and Methods

### 4.1. Cell Lines and Cell Culture

The PC3 human prostatic small cell carcinoma cell line [63] and the C4-2B cell line, which originated from the human adenocarcinoma LNCaP cell line after selection for metastasis to bone in mouse xenografts [64], were used in this study. Both cell lines were kindly provided by Charlotte Bevan (Imperial College London, London, UK). Cells were maintained in complete medium (RPMI 1640 with GlutaMAX^TM^ (Sigma-Aldrich Company Ltd., Gillingham, UK) supplemented with 10% Fetal Calf Serum (FCS) and 100 units/mL of penicillin and streptomycin (P/S)). Cells were cultured at 37 °C and 5% CO_2_.

### 4.2. Plasmid Vectors and gRNA Design

The sequences of the DKK3 gene promoter region containing the CpG islands were extracted using the UCSC genome browser. Potential gRNA sequences, predicted using sgRNA Designer (www.portals.broadinistitute.org), were selected to bind between 1 and 1000 bp upstream of the transcriptional start site (TSS). Five gRNAs targeting different DKK3 sites and a scrambled gRNA (gRNA-control) were cloned into pRP[gRNA]-Puro-U6 (Cyagen Biosciences Inc., Santa Clara, CA, USA). dCas9-VPR (#63798), dCas9-Tet1CD (#84475), and dCas9TetCD_IM (#84479) were from Addgene LGC Standards (Teddington, UK). Sequences for gRNAs are listed in Table S1.

### 4.3. Transient Transfection

Transfections were performed using the Lipofectamine^®^ LTX and Plus Reagent (Life Technologies Limited, Paisley, UK) according to the manufacturer’s protocol. Cells were transfected in 6-well plates seeded with 5 × 10^5^ cells per well. For experiments where gRNAs were transfected together, the amount of each gRNA was scaled down to equalize the total amount of gRNA used for each condition and the dCas9-VPR to gRNA ratio was 3:1.

### 4.4. RNA Extraction, cDNA Synthesis, and qPCR

RNA was isolated using TRIZOL reagent (Invitrogen, Carlsbad, CA, USA), according to manufacturer’s instructions. Quantitative real-time PCR (q-RT-PCR) was performed using the SYBR Green PCR Master Mix (Life Technologies Limited) and a 7900HT Fast Real-Time PCR thermal cycler (Fisher Scientific UK Ltd., Loughborough, UK), using the amplification parameters: 50 °C for 2 min and 95 °C for 10 min, followed by 40 cycles at 95 °C for 15 s and 60 °C for 1 min. Raw count values obtained with SDS 2.0 (Applied Biosystems, Foster City, CA, USA) were imported into Excel to calculate the fold changes, normalized to expression of the gene *36B4* in the same sample. Relative quantification was carried out using the 2-∆∆Ct method. The primer sequences and the concentrations used for PCR are in Table S2.

### 4.5. Demethylation Using 5-Aza-2′Deoxycytidine (5-Aza-dC)

PC3 cells were plated in 6-well plates for 24 h and treated with 1 μM 5-Aza-dC for three days, by which time cells were 80% confluent and then harvested. 5-Aza-dC was prepared fresh in ddH_2_O and filter-sterilized (Sigma-Aldrich Company Ltd.).

### 4.6. Genomic DNA Extraction, Bisulfite Conversion, and Combined Bisulfite Restriction Analysis (CoBRA)

Genomic DNA was extracted from PC3 cells using the DNeasy Blood and Tissue Kit (Qiagen Ltd., Manchester, UK). Bisulfite conversion of genomic DNA (500 ng) and methylated and unmethylated controls was carried out using the EZ DNA methylation kit (Zymo Research, Cambridge, UK) according to manufacturer’s instructions. PCR primers for the DKK3 promoter region were designed using MethPrimer software; primer sequences are in the Supplementary Data. The PCR amplification method was designed for bisulfite-modified DNA using Touch-Up gradient PCR [65]. A 10-cycle loop, consisting of 95 °C for 30 s, 48 °C for 30 s (−0.5 °C every cycle), and 72 °C for 1 min, was carried out and repeated five times (50 cycles). CoBRA amplified products were digested with BstUI (Life Technologies Limited) using 10 μL PCR product (0.1–0.5 μg of DNA) in a final volume of 25 μL with distilled water for 6 h at 37 °C.

### 4.7. Protein Extraction, Western Blotting, and ELISAs

Two days after cell transfection, media were changed to normal growth medium for cell extracts or serum-free medium for collection of cell-conditioned media (CM), supplemented with 1 μg/mL puromycin. Three days later, CM was collected from the cells cultured in serum-free media, and cells cultured in normal growth medium were washed with cold PBS and lysed in 300 μl RIPA (0.5% Na deoxycholate, 1% Triton X-100, 20 mM Tris pH 8.0, 0.1% SDS, 150 mM NaCl), with 1 mM EDTA, cOmplete^TM^ EDTA-free Protease Inhibitor Cocktail (Sigma-Aldrich Company Ltd.) and 1× PhosSTOP^TM^ Phosphatase Inhibitor Cocktail (Sigma-Aldrich Company Ltd.). Cells were then collected by scraping, transferred into a cold centrifuge tube and incubated on ice for 15 min. Lysates were centrifuged at 4 °C (15,000× *g*) for 15 min and the supernatant collected. Samples were separated by 6–8% SDS-PAGE and transferred to nitrocellulose or PVDF membranes (Bio-Rad Laboratories Ltd., Watford, UK). Membranes were blocked in 5% milk powder (Sigma) or 5% BSA (Sigma) in 1× Tris buffered saline, 0.1% Tween (TBST) for 1 h at room temperature. Blots were probed overnight with goat anti-DKK3 (1:500) at 4 °C. Membranes washed again 3 × 10 min in TBST and incubated with secondary antibodies (1:5000) for 1 h at room temperature, washed 3 × 5 min in TBST and then exposed to ECL Clarity reagent (BioRad), exposed to X-ray film, which were developed using an OptiMax film processor. Membranes were also probed with anti-human GAPDH (1:5,000) for 1 h at room temperature, and donkey anti-mouse-HRP linked secondary antibody (1:15,000) for 45 min. ELISAs were performed for CM from transfected cells using the Human Dkk-3 DuoSet kit (R&D Systems Ltd., Abingdon, UK), according to the manufacturer’s instructions. The absorbance of each standard and sample was determined using an OptiMax tunable microplate reader (Molecular Devices, San Jose, CA, USA) set to 450 nm and 540 nm. Samples were quantified based on a standard curve generated using recombinant purified Dkk-3 (R&D Systems) and a four-parameter logistic curve-fit.

### 4.8. Gene Reporter, Proliferation, and Migration Assays

Gene reporter assays were carried out using pGL3-CAGA12-luc and pRL-TK as previously described [24]. Briefly, 200,000 cells/well were plated in triplicate for each treatment in 12-well plates and transfected on the following day with gRNA, dCas9-VPR, 100 ng pRL-TK, and 400 ng CAGA-luc per well. After 5 h, an equal volume of medium containing 20% FCS and P/S was added. After 24 h, cells were treated with TGF-β1 (2 ng/mL) or the same volume of PBS. Cells were incubated for 24 h and then washed in cold PBS and lysed in 250 μL 1× passive lysis buffer (Promega UK, Southampton, UK), frozen and thawed, centrifuged at 15,000 rpm for 5 min and subjected to luciferase assays using the Dual-Glo^®^ luciferase assay kit (Promega), according to manufacturer’s instructions, using a Vector Light 1420 luminometer. For proliferation assays, cells were plated in triplicate for each time point (2 days: 200,000 cells/well; 5 days: 50,000 cells/well) in 12-well plates in complete RPMI medium. On the next morning, cells were transfected with gRNAs and dCas9-VPR as above. After 2 d or 5 d, cells were washed in PBS, fixed in ice-cold methanol (1 mL/well) for 20 min and allowed to dry for 30 min. Subsequent staining was performed using 0.2% crystal violet in 20% methanol (1 mL/well) for 20 min. A volume of 0.3 mL 10% acetic acid was added to the wells to elute the dye and 100 μL of the suspension was transferred to a 96-well plate. Optical density (OD) was measured in a plate reader at (Molecular Devices) at 595 nm. For migration assays, cells were plated in a 6-well plate at a density of 200,000 cells/well for the short time-point (2 d) or 50,000 cells/well for the long time-point (5 d). The day after plating, cells were transfected as above. For the short time point, cells were trypsinized after 24 h, while for the long time-point this was after 4 d. Cells were centrifuged at 500× *g* for 5 min and resuspended in 2 mL serum-free RPMI medium ± 2 ng/mL TGF-β1. 50,000 cells were plated on 8 μm pore Falcon Transwell^®^ polycarbonate membrane inserts (Scientific Laboratory Supplies Ltd., Nottingham, UK) in a 24-well plate, in duplicate for each treatment and transfection condition. 800 μL of complete medium was added to the outside of each insert. Cells were allowed to migrate for 24 h at 37 °C, and those that had not migrated were removed with a cotton bud. The inserts were washed in PBS and cells fixed in cold methanol (1 mL/insert) for 20 min, allowed to dry for 30 min and then stained with 0.2% crystal violet in 20% methanol (600 μL/insert) for 30 min. Inserts were washed in distilled water and seven pictures were taken for each insert at 200× magnification using a Nikon Eclipse TE200-U and QCapture software (Qimaging Inc., Surrey, BC, Canada). Migrated cells were counted using Image J and the average numbers of migrated cells calculated for each insert. This value was normalized by dividing the number of migrated cells by the OD reading at 595 nm from additional wells of cells that had been plated in parallel without inserts to control for effects on cell number.

### 4.9. Bioinformatics Analysis

MSKCC mRNA expression data were extracted from cBIO (www.cbioportal.org/) for selected genes using *z*-score vs normal with threshold ± 2. Oncomine (www.oncomine.org/) was also used to compare mRNA expression in prostate cancer datasets to determine the extent of their downregulation across multiple prostate cancer datasets. The differential expression of each gene between PCa and normal samples was analyzed for fold-change and statistical significance. ToppGene (www.toppgene.cchmc.org/) was used to investigate the relevance of the genes to the TGF-β signaling pathway, based on the functional similarity between the seed list and training gene list.

### 4.10. Statistical Analysis

All data are presented as mean and standard error of mean (SEM) or standard deviation (SD). Microsoft Excel was used to perform the two sample, paired Student’s *t*-test to calculate the statistical significance and to perform one-way ANOVA to compare multiple experimental conditions. A *p*-value < 0.05 was considered to be statistical significant. * *p* < 0.05. For qPCR analysis, ∆∆Ct values were log-transformed before the test.

## 5. Conclusions

In conclusion, in this study, we demonstrate that CRISPR-mediated activation of DKK3 expression is feasible, providing further evidence for potential therapeutic application of this technology, and have generated results strengthening the link between Dkk-3 and TGF-β regulation of gene expression.

## Supplementary Materials

The following are available online at http://www.mdpi.com/2072-6694/10/6/165/s1. Figure S1: Methodological workflow of analyses performed; Figure S2: Analysis of the expression levels of the indicated genes in PCa datasets; Figure S3: Location of CpG probes in the DKK3 promoter; Figure S4: Effects of TGF-β and CRISPR induction of DKK3 on *DNMT* mRNA expression; Table S1: Sequences and genomic coordinates of gRNAs used to target the DKK3 promoter; Table S2: Primer sequences used for qPCR AND CoBRA
