# Supplementary material for: CRISPR-Mediated Reactivation of DKK3 Expression Attenuates TGF-β Signaling in Prostate Cancer
Source: Cancers (Basel). 2018 May 28;10(6):165. doi: 10.3390/cancers10060165 (PMC6025141; doi:10.3390/cancers10060165)
Supplement: Supplementary file 1 [file cancers-10-00165-s001.pdf]

Supplementary Materials: CRISPR-mediated reactivation of DKK3 expression attenuates TGF-signaling in prostate cancer

Hoda Kardooni, Estela Gonzalez-Gualda, Emmanouil Stylianakis, Sina Saffaran, Jonathan Waxman and Robert M. Kypta

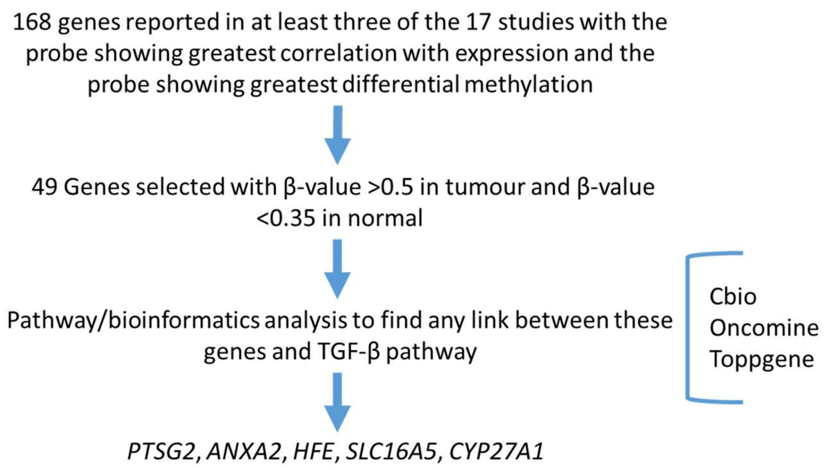

Figure S1. Methodological workflow of analyses performed

Gene symbol	Oncomine gene expression	MSKCC 2010 Gene Expression (cBIO)
CDKN2A	2 down	
HFE	6 down	
PDX1		
PTGS2	5 down	
MSX1		
ANXA2	12 down	
NKX2-1	3 down	
EFEMP1	1 down	
PAX5		
SOX17		
LMX1B		
PROM1	8 down	
SIX6	1 down	
CRHBP	2 down	
HES5	1 down	
TBX15		
NKX2-2		
HIST1H4F		
UCN	1 down	
VSX1	2 down	
TNFRSF10C	5 down	
VAX1		
CDX4	1 down	
CYP27A1	8 down	
RND2	5 down	
EN2	1 down	
HOXC4		
DAB1	3 down	
WNT16		
NKX2-3		
POU3F3		
LHX9		
ZNF154	2 down	
GSX2		
HS3ST1	1 down	
TMEM176B	1 down	
OTP	2 down	
GPR50	1 down	
AKAP2	1 down	
RHCG		
LAMP5		
SPATA6	3 down	
fOXB2		
SLC16A5	9 down	
MOB3B		
WT1-AS		
CCDC181	1 down (C1orf114)	
TMEM106A	2 down	
WDR86		

Figure S2. Analysis of the expression levels of the indicated genes in PCa datasets. Bars on right indicate upregulation (red), no change (grey) and downregulation (blue) in tumors.

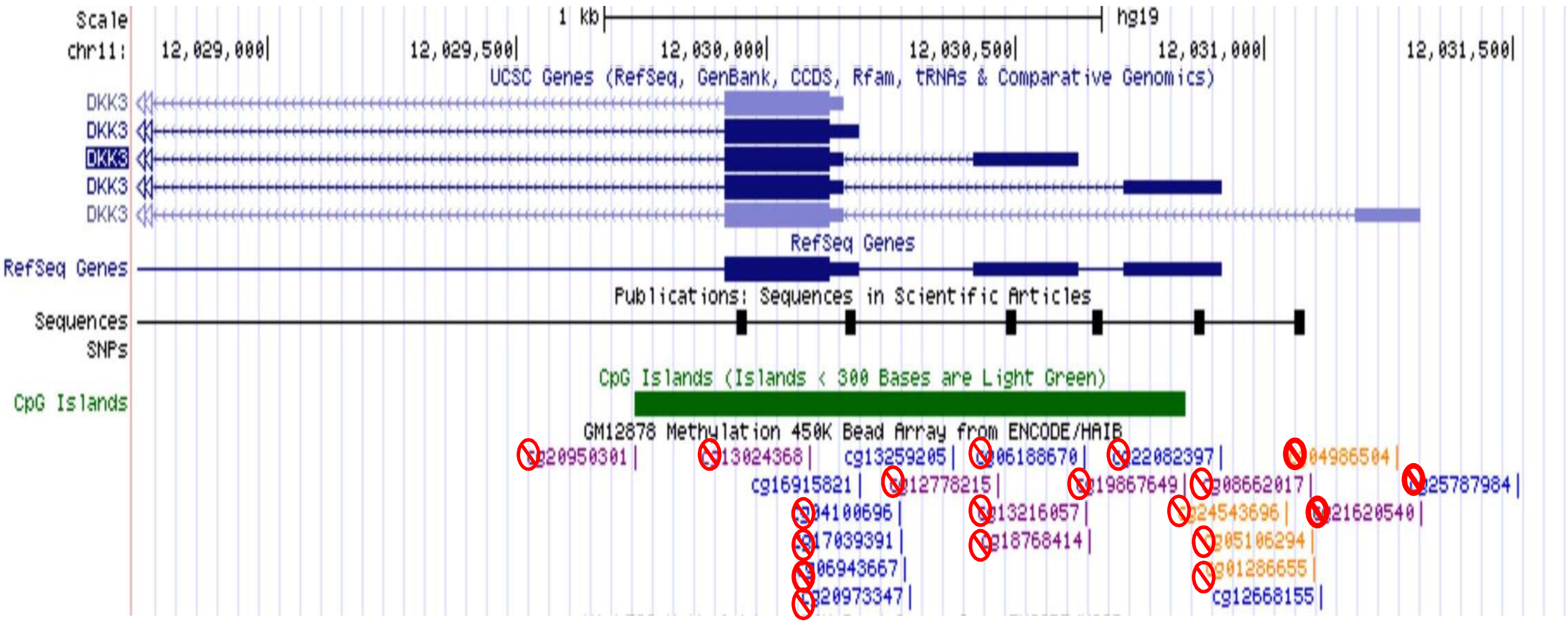

Figure S3. Location of CpG probes in the DKK3 promoter

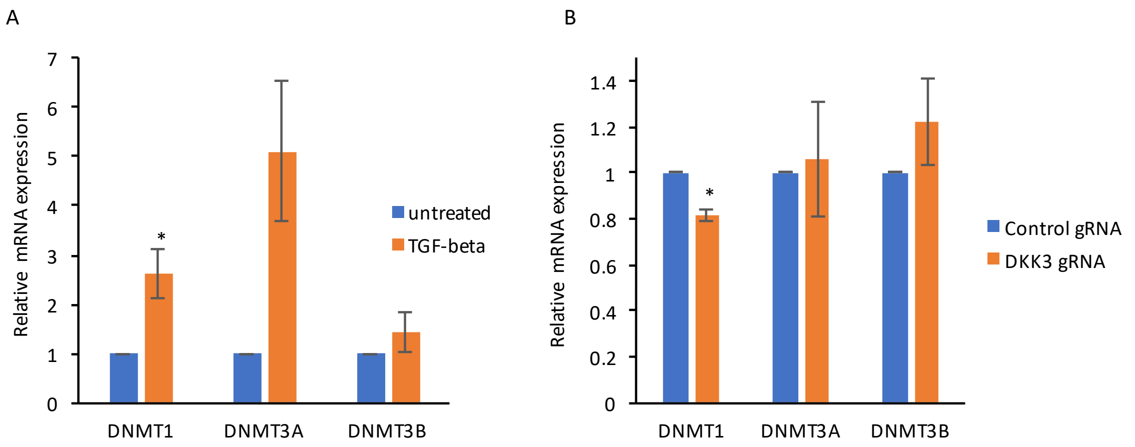

Figure S4. Effects of TGF-b and CRISPR induction of DKK3 on *DNMT* mRNA expression. Q-RT-PCR analysis of the indicated genes in PC3 cells, either untreated and treated with TGF-b for 24 h (A) or transfected with dCas9-VPR and either control gRNA or DKK3 gRNAs (B). Graphs show mRNA expression fold change ± SD, versus control treatment, $n = 3$, $*p < 0.05$ two-tailed Student's t-test.

Table S1. Sequences and genomic coordinates of gRNAs used to target the DKK3 promoter

gRNA	gRNA sequence	Genomic Coordinates
1	GTGGCGGTAAACAGTAATGTG	chr11:12030012-12030032
2	GTCTGCCCGAAGTGACAAGCG	chr11:12030132-12030152
3	GAGACGGGCCTGGGATGCCGC	chr11:12029712-12029732
4	GCAACTCGGTCCAGTCGGGGT	chr11:12029472-12029492
5	GTTCCCGCACCCGCCCGGAGA	chr11: 12029168 -12029187
control	TACCAGAGCTAACTCA	-

Table S2. Primer sequences used for qPCR AND CoBRA

Gene name	Forward Primer (F) (5′ → 3′)	Reverse Primer (R) (5′ → 3′)	Primer concentration (F/R) nM
DKK3	TCATCACCTGGGAGCTAGAG	TTCATACTCATCGGGGACCT	500/500
36B4	GTGTTCGACAATGGCAGCAT	AGACACTGGCAACATTGCGGA	500/500
ANXA2	CTCTACACCCCCAAGTGCAT	TCAGTGCTGATGCAAGTTCC	300/300
HFE	CCTTGTTTGAAGCTTTGGGC	CACGGCGACTCTCATGATCA	300/600
PTGS2	TGCCATTCTTTGCCCAGCACT	AAAGGCGCAGTTTACGCTGT	600/600
SLC16A5	GCCCTGCTTGAGTCTGGAATG	ACTGCCAATGTGGCTGCTG	900/900
ACGT2	TACCCCATTGAACACGGCAT	TGCTCTTCAGGTGCTACACG	600/600
FZD8	TAGAGCTAGAAATAGCAAGT	GCCACTTTTTCAAGTT	600/600
TGFBI	CACCAAGAGAACGGAGCAGA	GCCTCCGCTAACCAGGATTT	300/300
ECM1	ATTTGGCTGTTGCTTCTGCT	TCTTGAAAGTGCTCTGGCCT	600/600
CYP27A1	GTGTCTGGCTACCTGCACTT	TTGGATGTCGTGTCCACTCC	600/600
NKD1	ACTTCCAGCCGAAAGTCGT	CACCATAGGCCGAAGCAC	900/900
DNMT1	CGACTACATCAAAGGCAGCAACCTG	TGGAGTGGACTTGTGGGTGTTCTC	300/900
DNMT3A	CGAGTCCAACCCTGTGATGATTG	GCTGGTCTTTGCCCTGCTTTATG	600/600
DNMT3B	TTGGAATAGGGGACCTCGTGTG	AGAGACCTCGGAGAACTTGCCATC	600/600
DKK3 (CoBRA)	TGGGTTGTTGTAAGTTTGAAGGT	CTCACCCACCCCTACTAAAC	500/500

© 2018 by the authors. Licensee MDPI, Basel, Switzerland. This article is an open access article distributed under the terms and conditions of the Creative Commons Attribution (CC BY) license (http://creativecommons.org/licenses/by/4.0/).
